# Presentation, Outcomes, and Non-elective Surgical Management of Diverticulitis: Is There an Ethnic Divide?

**DOI:** 10.1007/s11605-023-05638-4

**Published:** 2023-02-28

**Authors:** Caitlyn Braschi, Jessica K. Liu, Ashkan Moazzez, Beverley A. Petrie

**Affiliations:** 1 Division of Colon & Rectal Surgery, Department of Surgery, Harbor-UCLA Medical Center, Torrance, USA; 2 Division of General & Bariatric Surgery, Department of Surgery, Harbor-UCLA Medical Center, 1000 W. Carson Street, Bldg F10, Torrance, CA 90505 USA

**Keywords:** Diverticulitis, Health disparities, Ethnic disparities, Colon surgery

## Introduction

The Hispanic population is an important and rapidly growing minority group in the USA that is relatively understudied in surgical literature. Hispanics have been found to live longer than non-Hispanics, and in some cases have better health outcomes despite higher risk profiles, a concept known as the Hispanic epidemiologic paradox.^1^ However, there also is contradicting literature showing worse health outcomes in this ethnic minority group.^2^ The prevalence of diverticulitis within the Hispanic ethnicity group is comparable to that of non-Hispanic Whites making it important to understand the impact of this common disease on outcomes specific to ethnicity. This study aims to compare the presentation, outcomes, and non-elective surgical management of diverticulitis in Hispanic versus non-Hispanic patients.

## Materials and Methods

The American College of Surgeons National Surgical Quality Improvement Program (ACS NSQIP) database was used to identify adult patients with acute diverticulitis (International Classification of Diseases, 10th revision codes K57.20 and K57.32) who underwent non-elective surgery (Current Procedural Terminology codes 44140–6, 44160, and 44204–8) during the index admission from 2015 to 2019. Ethnicity was defined as either Hispanic or non-Hispanic, consistent with most standard databases including the US Census. Ethnicity refers to the shared culture of a group from a similar region, distinct from race which is ascribed based more on physical characteristics and shared history. Both variables were included in the analysis along with comorbidities and perioperative characteristics. Diverticulitis was defined as either complicated (abscess or perforation) or uncomplicated. Thirty-day outcomes were compared between ethnic groups. This study was approved by the Institutional Review Board.

## Results

A total of 13,095 patients were included in the study, of which 46.8% were male with a mean age of 61.3 ± 14.2 years. A small proportion (8.3%) of patients were of Hispanic ethnicity. Hispanic patients were younger (52.9 vs 62.1 years, *p* < 0.001), had lower rates of several comorbidities (see Table 1), and were significantly less likely to present with complicated disease (83.2% vs 86.4%, *p* = 0.003) and preoperative sepsis (47.4% vs 51.2%, *p* = 0.019). In bivariate analysis, 30-day mortality was lower among Hispanic patients (*p* < 0.001) but there was no difference in overall 30-day morbidity by ethnicity (see Table 2). In multivariate regression analysis, Hispanic ethnicity was independently associated with lower 30-day mortality (OR 0.447, 95% CI 0.210–0.951, *p* = 0.037) (see Table 3). The Hosmer and Lemeshow test was not significant, confirming the model was a good fit.

**Table 1:** Baseline and perioperative characteristics of patients who underwent non-elective surgery for acute diverticulitis by ethnicity

Characteristic	Non-Hispanic (*N* = 12,007)	Hispanic (*N* = 1088)	*p*-value
Age	62.1 (± 13.7)	52.9 (± 15.7)	< 0.001
Sex, male	5520 (46.0%)	614 (56.4%)	< 0.001
Race			< 0.001
White	10,518 (89.1%)	773 (97.5%)	–
Black	1033 (8.8%)	13 (1.6%)	–
Asian	146 (1.2%)	1 (0.1%)	–
American Indian/Alaska Native	79 (0.7%)	2 (0.3%)	–
Native Hawaiian/Pacific Islander	24 (0.2%)	4 (0.5%)	–
BMI	30.0 (± 7.3)	31.6 (± 7.6)	< 0.001
Diabetes mellitus	1314 (10.9%)	163 (15.0%)	< 0.001
Hypertension	6142 (51.2%)	399 (36.7%)	< 0.001
CHF	240 (2.0%)	14 (1.3%)	0.108
COPD	989 (8.2%)	24 (2.2%)	< 0.001
Tobacco use	2908 (25.2%)	160 (20.4%)	0.002
Dialysis dependence	179 (1.5%)	15 (1.4%)	0.797
Chronic steroid use	1328 (11.1%)	62 (5.7%)	< 0.001
Acute kidney injury	122 (1.0%)	11 (1.0%)	1.000
Bleeding disorder	1129 (9.4%)	53 (4.9%)	< 0.001
Metastatic cancer	305 (2.5%)	11 (1.0%)	0.002
Ascites	94 (0.8%)	11 (1.0%)	0.475
Weight loss	556 (4.6%)	41 (3.8%)	0.198
ASA Class 1 or 2	3844 (32.0%)	461 (42.4%)	< 0.001
Preop transfusion	192 (1.6%)	20 (1.8%)	0.615
Preop wound infection	390 (3.2%)	17 (1.6%)	0.003
Preop sepsis	6145 (51.2%)	516 (47.4%)	0.019
Preop ventilator	115 (1.0%)	6 (0.6%)	0.191
Wound class			0.001
Clean	55 (0.5%)	7 (0.6%)	–
Clean-contaminated	1729 (14.4%)	178 (16.4%)	–
Contaminated	1630 (13.6%)	184 (16.9%)	–
Dirty	8593 (71.6%)	719 (66.1%)	–
Complicated disease	10,373 (86.4%)	905 (83.2%)	0.003

Categorical variables presented as *N* (%). Continuous variables presented as mean (± standard deviation)

*ASA*, American Society of Anesthesiologists; *BMI*, body mass index; *CHF*, congestive heart failure; *COPD*, chronic obstructive pulmonary disease

**Table 2:** Bivariate results of short-term post-operative outcomes by ethnicity

	*p*-value	Odds ratio	95% C.I	
			Lower	Upper
30-day mortality	< 0.001	0.305	0.179	0.521
30-day morbidity	0.248	0.929	0.819	1.053
SSI (any)	0.249	0.905	0.765	1.072
Superficial SSI	0.247	1.166	0.248	0.899
Deep SSI	0.399	1.270	0.728	2.216
Organ space SSI	0.042	0.808	0.657	0.993
Wound dehiscence	0.049	0.584	0.340	1.004
Pneumonia	< 0.001	0.450	0.298	0.680
Reintubation	< 0.001	0.336	0.193	0.586
Pulmonary embolism	0.715	0.892	0.481	1.653
Prolonged ventilator	0.005	0.642	0.470	0.875
Renal insufficiency	0.759	0.904	0.473	1.727
Renal failure	0.029	0.413	0.182	0.936
Urinary tract infection	0.979	0.994	0.619	1.596
Stroke	0.031*	–	–	–
Cardiac arrest	0.007*	0.255	0.081	0.801
Myocardial infarction	0.005*	0.223	0.071	0.701
DVT	0.027	0.569	0.343	0.944
Post-op sepsis	0.001	1.273	1.103	1.469
Post-op septic shock	< 0.001	0.571	0.436	0.748
Post-op transfusion	< 0.001	0.630	0.498	0.797
Laparoscopic surgery	< 0.001	1.500	1.305	1.725
Ostomy creation	0.004	0.823	0.720	0.941
Operation duration, min	0.284	− 4.931	− 9.926	0.063
LOS, days	0.008	0.814	0.335	1.293
Reoperation	0.051	0.757	0.572	1.002
Readmission	0.840	0.978	0.801	1.194

Categorical variables presented as *N* (%). Continuous variables presented as mean (± standard deviation)

*DVT*, deep venous thromboembolism; *LOS*, length of stay; *SSI*, surgical site infection

* Fisher’s Exact test

**Table 3:** Multivariate regression of factors significantly associated with 30-day mortality

	*p*-value	Odds ratio	95% C.I. for odds ratio	
			Lower	Upper
Age	< 0.001	1.085	1.073	1.097
Hispanic ethnicity	0.032	0.438	0.206	0.933
Native Hawaiian/Pacific Islander race	0.026	4.954	1.209	20.302
Diabetes mellitus	0.012	1.405	1.079	1.829
COPD	< 0.001	2.232	1.732	2.878
Ascites	< 0.001	3.699	1.938	7.059
CHF	< 0.001	2.325	1.571	3.441
Preop ventilator dependence	< 0.001	4.139	2.535	6.758
Dialysis dependence	< 0.001	2.654	1.539	4.272
Metastatic cancer	< 0.001	5.085	3.598	7.188
Chronic steroid use	< 0.001	1.571	1.234	2.001
Bleeding disorder	< 0.001	1.850	1.455	2.351
Preoperative transfusion	0.040	1.699	1.025	2.816
Preoperative wound infection	0.001	1.892	1.281	2.794
Preoperative sepsis	< 0.001	2.728	2.115	3.517
Emergent surgery	0.001	1.509	1.195	1.904
Wound classification	0.049	1.210	1.001	1.463
Laparoscopic surgery	0.046	0.696	0.487	0.994

*CHF*, congestive heart failure; *COPD*, chronic obstructive pulmonary disease

## Discussion

This study found no difference in overall 30-day morbidity among Hispanic versus non-Hispanic patients who underwent surgery for acute diverticulitis, whereas 30-day mortality was lower among Hispanic patients compared to non-Hispanic patients. In addition, Hispanic patients had other favorable outcomes including shorter lengths of stay, higher rates of minimally invasive surgery, and fewer ostomy creations.

These results were surprising given the inequities in access to care and socioeconomic status of Hispanics in the USA. Hispanic patients in this dataset did have some favorable baseline features including fewer comorbidities, less complicated disease, and less preoperative sepsis which could explain higher rates of laparoscopy and lower stoma creation in this group. Prior studies using NSQIP data have found similar outcomes among Hispanic patients,^3,4^ the largest of which included 3.5 million patients, and found that Hispanic patients had lower 30-day post-operative morbidity and mortality.^5^ Another consideration to support these findings is the Hispanic epidemiological paradox, driven by factors that are not fully understood.^5^ Additionally, Hispanic ethnicity itself is a widely diverse group.^6^

This study suggests that Hispanic patients actually fare better compared to non-Hispanic patients undergoing non-elective surgery for acute diverticulitis. Future research should investigate the specific factors driving these differences in outcomes and the surgical outcomes for subgroups within the Hispanic population.

## Data Availability

These data are avaialble by request from the American College of Surgeons National Surgical Quality Improvement Program.
